# Supplementary material for: The Role of Paraclinical Investigations in Detecting Inflammation in Children and Adolescents with Obesity and Metabolic Syndrome
Source: Life (Basel). 2024 Sep 23;14(9):1206. doi: 10.3390/life14091206 (PMC11433473; doi:10.3390/life14091206)
Supplement: Supplementary file 1 [file life-14-01206-s001.zip › life-3204903-supplementary.pdf]

Table S1. Descriptive statistic of MetS parameters depending on MetS presence and MetS diagnosis criteria.

			All subjects				MetS- group				MetS+group			
			Mean	Median	Percentile 25	Percentile 75	Mean	Median	Percentile 25	Percentile 75	Mean	Median	Percentile 25	Percentile 75
Age (years)			11	11	9	13	11_a	11	9	12	11_a	11	10	14
BMI (kg/m2)			28.151	27.476	25.848	30.586	27.910_a	27.476	25.475	30.586	28.445_a	27.452	26.008	30.642
SBP	SBP<90 percentile	SBP (mmHg)	108	110	102	115	107_a	107	100	115	110_a	111	102	115
	SBP>90 percentile	SBP (mmHg)	129	130	120	136	127_a	129	120	134	130_a	130	120	139
DBP	DBP<90 percentile	DBP (mmHg)	67	68	63	74	68_a	70	64	73	66_a	65	59	74
	DBP>90 percentile	DBP (mmHg)	85	85	80	90	88_a	86	83	95	84_a	84	78	87
Glycemia	Glycemia<100mg/dL	Glycemia (mg/dL)	85	85	80	91	87_a	88	81	93	83_a	84	78	89
	Glycemia>100mg/dL	Glycemia (mg/dL)	115	105	103	131	.[1]				115[1]	105	103	131
Triglyceride	Triglyceride<110 mg/dL	Triglyceride (mg/dL)	69.76	70.00	53.00	87.00	67.89_a	66.50	52.00	86.00	74.92_a	76.00	60.00	89.00
	Triglyceride>110 mg/dL	Triglyceride (mg/dL)	175.14	155.00	129.00	191.00	185.00_a	186.00	129.00	240.00	173.58_a	155.00	129.00	191.00
HDL-c	HDL-c>40mg/dL	HDL-c (mg/dL)	50.37	47.24	44.96	54.47	51.34_a	49.00	44.99	57.50	45.91_a	46.23	43.49	47.24
	HDL-c<40mg/dL	HDL-c (mg/dL)	32.64	34.55	30.41	37.07	35.19_a	34.98	32.84	39.00	31.92_a	34.50	30.12	35.87
Total cholesterol (mg/dL)			169.0	159.0	143.0	198.0	166.8_a	160.0	150.6	184.0	171.7_a	158.5	139.5	210.0

Note: Values in the same row and subtable not sharing the same subscript are significantly different at $p < 0.05$ in the two-sided test of equality for column means. Cells with no subscript are not included in the test. Tests assume equal variances.[2]

[1.] This category is not used in comparisons because there are no other valid categories to compare

[2.] Tests are adjusted for all pairwise comparisons within a row of each innermost subtable using the Bonferroni correction.

Table S2. Descriptive statistics of the clinical and paraclinical parameters according to the presence or absence of MetS.

	All subjects				MetS- group				MetS+group			
	Mean	Median	Percentile 25	Percentile 75	Mean	Median	Percentile 25	Percentile 75	Mean	Median	Percentile 25	Percentile 75
**Age (years)**	11	11	9	13	11_a	11	9	12	11_a	11	10	14
**BMI (kg/m2)**	28.151	27.476	25.848	30.586	27.910_a	27.476	25.475	30.586	28.445_a	27.452	26.008	30.642
**SBP (mmHg)**	117	115	107	128	114_a	112	105	120	121_b	120	111	131
**DBP (mmHg)**	75	75	66	85	73_a	72	66	80	78_a	80	72	85
**Glycemia (mg/dL)**	89	88	81	95	87_a	88	81	93	93_a	89	82	102
**Triglyceride (mg/dL)**	102.41	85.00	60.00	129.00	76.90_a	69.00	53.00	88.00	133.50_b	123.00	79.00	159.50
**HDL-c (mg/dL)**	42.38	41.41	34.64	48.00	48.44_a	46.62	41.41	54.47	34.98_b	35.28	31.17	39.35
**Total cholesterol (mg/dL)**	169.0	159.0	143.0	198.0	166.8_a	160.0	150.6	184.0	171.7_a	158.5	139.5	210.0

Note: Values in the same row and subtable not sharing the same subscript are significantly different at $p < 0.05$ in the two-sided test of equality for column means. Cells with no subscript are not included in the test. Tests assume equal variances.[1]

[1.] Tests are adjusted for all pairwise comparisons within a row of each innermost subtable using the Bonferroni correction.

Table S3. Clinical and paraclinical diagnostic criteria values categorized by the number of MetS criteria met.

		Mean	Median	Percentile 25	Percentile 75
1 MetS criteria	BMI (kg/m2)	27.805	27.579	25.626	29.692
	SBP (mmHg)	108	109	100	115
	DBP (mmHg)	69	69	66	74
	Glycemia (mg/dL)	87	89	81	93
	Triglyceride (mg/dL)	62.94	60.50	49.50	76.00
	HDL-c (mg/dL)	54.05	53.16	46.91	59.06
	Total cholesterol (mg/dL)	175.2	166.0	152.5	198.5
2 MetS criterias	BMI (kg/m2)	27.983	27.271	25.182	30.664
	SBP (mmHg)	118	115	105	130
	DBP (mmHg)	76	74	66	85
	Glycemia (mg/dL)	87	88	81	93
	Triglyceride (mg/dL)	86.61	83.00	60.00	95.00
	HDL-c (mg/dL)	44.54	43.27	39.00	47.92
	Total cholesterol (mg/dL)	161.0	159.0	142.0	176.0
3 MetS criterias	BMI (kg/m2)	28.254	28.033	24.880	30.121
	SBP (mmHg)	119	115	110	126
	DBP (mmHg)	78	80	69	85
	Glycemia (mg/dL)	86	85	79	91
	Triglyceride (mg/dL)	105.45	96.50	75.50	132.00
	HDL-c (mg/dL)	38.70	38.02	34.55	44.46
	Total cholesterol (mg/dL)	171.3	157.0	139.5	217.5
4 MetS criterias	BMI (kg/m2)	27.062	26.175	25.911	28.887
	SBP (mmHg)	122	123	112	130
	DBP (mmHg)	76	78	67	85
	Glycemia (mg/dL)	92	95	82	104
	Triglyceride (mg/dL)	174.29	166.00	99.00	287.00
	HDL-c (mg/dL)	29.58	31.64	24.81	35.55
	Total cholesterol (mg/dL)	172.6	159.0	133.0	207.0
5 MetS criterias	BMI (kg/m2)	31.145	31.673	26.741	34.781
	SBP (mmHg)	131	130	125	139
	DBP (mmHg)	81	76	76	86
	Glycemia (mg/dL)	120	121	103	131
	Triglyceride (mg/dL)	188.60	155.00	155.00	249.00
	HDL-c (mg/dL)	27.68	30.12	28.46	30.69
	Total cholesterol (mg/dL)	172.0	180.0	149.0	206.0

Table S4. CBC parameters, inflammatory and cardioembolic indexes according to the presence or absence of MetS.

	MetS- group				MetS+ group				
	**Mean**	**Median**	**Percentile 25**	**Percentile 75**	**Mean**	**Median**	**Percentile 25**	**Percentile 75**	**p-value**
**ESR (mm/houe)**	16_a	13	8	18	21_a	18	11	28	0.114
**#leukocytes**	8.079_a	7.590	6.330	9.770	8.160_a	8.204	6.719	9.510	0.694
**#lymphocytes**	3.023_a	3.069	2.200	3.510	2.949_a	2.789	2.357	3.455	0.6
**#neutrophils**	4.214_a	3.800	3.103	5.100	4.390_a	4.235	3.091	5	0.583
**#monocytes**	$.590_a$	0.580	0.470	0.700	$.567_a$	0.499	0.415	0.679	0.392
**#platelets**	323.0_a	306.0	263.8	374.7	327.2_a	312.9	286.9	382.450	0.470
**#basophiles**	$.090_a$	0.099	0.050	0.108	$.078_a$	0.071	0.052	0.100	0.286
**NLR**	1.455_a	1.439	1.003	1.803	1.538_a	1.561	1.084	1.783	0.552
**PLR**	112.582_a	107.279	85.207	134.667	117.653_a	114.217	94.760	137.2	0.474
**SII**	465.032_a	433.571	313.943	534.650	510.985_a	493.400	308.870	592.336	0.425
**TG/HDL-c**	1.681_a	1.559	1.117	1.955	4.304_b	3.231	2.252	4.974	0.000
**Atherogenic index of plasma**	$.173_a$	0.193	0.048	0.291	$.548_b$	0.509	0.353	0.697	0.000
**TC/HDL-C**	3.541_a	3.563	2.827	4.140	5.335_b	4.824	3.994	6.038	0.0
**MHR**	$.013_a$	0.012	0.009	0.015	$.018_b$	0.016	0.012	0.021	0.015
**LHR**	$.065_a$	0.064	0.051	0.078	$.095_b$	0.084	0.065	0.101	0.001
**NHR**	$.032_a$	0.029	0.020	0.039	$.048_b$	0.044	0.034	0.055	0.001
**PHR**	6.972_a	6.548	5.433	8.334	10.371_b	9.040	7.929	10.585	<0.001
**non-HDL-c**	118.37_a	116.51	97.02	129.13	136.67_b	122.46	102.50	175.765	0.071

Note: Values in the same row and subtable not sharing the same subscript are significantly different at $p < 0.05$ in the two-sided test of equality for column means. Cells with no subscript are not included in the test. Tests assume equal variances.[1]

1. Tests are adjusted for all pairwise comparisons within a row of each innermost subtable using the Bonferroni correction.
